# Integrating care for individuals with FASD: results from a multi-stakeholder symposium

**DOI:** 10.1186/s12913-015-1113-8

**Published:** 2015-10-05

**Authors:** Paul Masotti, Sally Longstaffe, Holly Gammon, Jill Isbister, Breann Maxwell, Ana Hanlon-Dearman

**Affiliations:** Community Wellness Department, Native American Health Center, 3124 International Blvd. Ste 214, Oakland, CA 94601 USA; Children’s Hospital of Winnipeg, University of Manitoba, CK253-840 Sherbrook Street, Winnipeg, Manitoba R3A 1S1 Canada; Healthy Child Manitoba Office, Government of Manitoba, 3rd Floor - 332 Bannatyne Avenue, Winnipeg, Manitoba R3A 0E2 Canada; Manitoba FASD Centre, 633 Wellington Crescent, Winnipeg, Manitoba R3M 0A8 Canada

**Keywords:** Fetal alcohol spectrum disorder, Integrating care, Primary care, Health policy, Research needs

## Abstract

**Background:**

Fetal Alcohol Spectrum Disorder (FASD) has a significant impact on communities and systems such as health, education, justice and social services. FASD is a complex neurodevelopmental disorder that results in permanent disabilities and associated service needs that change across affected individuals’ lifespans. There is a degree of interdependency among medical and non-medical providers across these systems that do not frequently meet or plan a coordinated continuum of care. Improving overall care integration will increase provider-specific and system capacity, satisfaction, quality of life and outcomes.

**Methods:**

We conducted a consensus generating symposium comprised of 60 experts from different stakeholder groups: Allied & Mental Health, Education, First Nations & Métis Health, Advocates, Primary Care, Government Health Policy, Regional FASD Coordinators, Social Services, and Youth Justice. Research questions addressed barriers and solutions to integration across systems and group-specific and system-wide research priorities. Solutions and consensus on prioritized lists were generated by combining the Electronic Meeting System approach with a modified ‘Nominal Group Technique’.

**Results:**

FASD capacity (e.g., training, education, awareness) needs to be increased in both medical and non-medical providers. Outcomes and integration will be improved by implementing: multidisciplinary primary care group practice models, FASD system navigators/advocates, and patient centred medical homes. Electronic medical records that are accessible to multiple medical and non-medical providers are a key tool to enhancing integration and quality. Eligibility criteria for services are a main barrier to integration across systems. There is a need for culturally and community-specific approaches for First Nations communities.

**Conclusions:**

There is a need to better integrate care for individuals and families living with FASD. Primary Care is well positioned to play a central and important role in facilitating and supporting increased integration. Research is needed to better address best practices (e.g., interventions, supports and programs) and long-term individual and family outcomes following a diagnosis of FASD.

## Background

Internationally, Fetal Alcohol Spectrum Disorder (FASD) has emerged as having a significant impact on communities and systems within communities such as health (e.g., primary care, acute care and specialty care), education, justice and social services [1]. FASD is a complex neurodevelopmental disorder that results in permanent disabilities [1, 2]. The cognitive and behavioural disability-related profiles and subsequent service needs of affected individuals change across their lifespans [1]. Lack of awareness of FASD and diagnostic capacity has contributed to FASD being under or misdiagnosed [3]. As a result, individuals affected with FASD are frequently identified later in life because of problems and needs identified by the different systems.

There is a degree of interdependency among medical and non-medical providers across these systems. Improving overall care integration (e.g., information sharing, communication, coordination, awareness and education) will increase provider-specific and overall system capacity. This increased capacity will translate into a decreased impact of FASD (e.g., human health, secondary disabilities and economic), increased continuity of care, quality of life for individuals and families living with FASD, and increased provider satisfaction [4–8].

This article describes the results of a Canadian Institutes of Health Research funded symposium that was attended by medical and non-medical care providers from various sectors who were gathered to address the issue of integrating care for individuals with FASD. Participants were considered leaders in their fields with expertise and experience working with individuals and families living with FASD.

### FASD – The Health Issue

Fetal Alcohol Spectrum Disorder is the leading cause of developmental disabilities in Canada and is the result of prenatal exposure to alcohol [9, 10]. Permanent effects impact the physical, mental, and emotional health of individuals, family and community. The further impact of the associated cognitive and behavioural disabilities frequently leads to secondary disabilities such as school withdrawal, substance abuse, and involvement with the legal system [2, 11, 12]. A diagnosis of FASD typically requires a comprehensive neurodevelopmental assessment of multiple domains of brain function (See Table 1) to evaluate the extent of the brain injury caused by prenatal alcohol exposure [2]. The specific range of impairments is influenced by multiple factors including timing and amount of alcohol exposure. The result is a complex disability which is unique to each individual but where permanent neurodevelopmental impairment and adaptive disabilities are the norm. Consequently, the care of individuals with FASD frequently requires a life-time of services from multi-sectoral medical and non-medical providers or agencies that do not frequently meet or plan a coordinated continuum of care.

**Table 1:** Domains of brain function and disabilities associated with prenatal alcohol exposure

Domain	Characteristics and Commonly Associated Disabilities
Physical Motor Skills	Gross and fine motor skills. Poor hand/eye coordination and sensory input. Abnormal muscle tone effects balance. Children may demonstrate problems or be developmentally delayed with simple tasks such as using scissors or pencils.
Sensory Processing Skills	Problems processing and interpreting sensory information (e.g., touch, sound, movement). Often are oversensitive resulting in over stimulation which leads to anxiety, aggressive behaviour and inability to learn or perform.
Cognition	Knowing, perception, awareness and judgement. Problems include: learning difficulties, deficits in math and school performance, poor impulse control, social perception, poor capacity for abstract thinking, and problems with memory, attention, judgement or organization.
Communication	Includes both expressive and receptive communication skills. May have problems with: using complex language structures, retrieving words from memory, following instructions, comprehension, discrimination, generalization, abstraction, and sequencing.
Academic Achievement	Multiple deficits impact academic achievement in multiple areas. However, children may excel in one area but be poor in another.
Memory	Problems with encoding, storage and retrieval. At times, may not be able to complete a task that has been successfully completed many times before.
Executive Functioning Abstract Reasoning	Includes higher order cognitive processes: inhibition, flexibility, cause and effect, judgment and organization. May show poor ‘common sense’ and ability to learn from the past or generalize.
Attention Deficit/Hyperactivity	Difficulty maintaining attention, easily distracted by visual and auditory stimulation and may have problems self-regulating when they are overstimulated or tired.
Adaptive Behaviour [Chudley et al., 2005]	Includes functioning independently and acquiring new daily living skills. Children have decreased capacity to develop/acquire new social, practical and conceptual skills to help them better respond to daily demands.

### What is the impact of FASD?

Health Canada describes FASD as a national public health, education, economic and societal concern [13]. In addition to the high human health costs (indicated above), FASD also has high societal and economic costs associated with both direct service needs and secondary disabilities. For example, in a study of 471 adults and 804 children in Alberta, Thanh et al., estimated the annual cost of secondary disabilities, if no network was in place throughout the province, would be $22.85 million (including $8.62 million for adults and $14.24 million for children) per year [14]. A 2009 Canadian study by Stade et al., evaluated the cost of FASD from birth to age 53 years and reported the adjusted annual individual costs to be $21,642 [95 % CI: $19,842-$24,041] which translated into an annual total Canadian cost of $5.3 Billion [95 % CI: $4.12 - $6.4B] [15].

FASD has also been described as a ‘hidden disability’ because there is often no identifying physical characteristics and behaviours are often the only symptoms [16]. Furthermore, an accurate understanding of the prevalence of prenatal exposure to alcohol is difficult because no reliable biological markers exist to identify those prenatally exposed [2]. Thus, we do not have accurate national estimates of the FASD prevalence in Canada and the commonly cited Health Canada estimate of 9.1 per 1000 births is extrapolated from older American studies [13]. However, FASD is widely considered to be underdiagnosed and we know the risk is high [16, 17]. For example, 74 % of Canadian women drink alcohol and many pregnancies are unplanned which means that women who regularly drink are frequently 3–4 weeks or possibly more post conception before they may be aware of their pregnancy [18].

Recent school studies in Canada and the United States have reported higher FASD prevalence rates. Thanh et al., reported a 11.7 prevalence rate in Alberta [19]. May et al., [20] reported a 3.6 % rate in a sample of 2,033 first grade students in a Midwestern US city and previous studies in the United States, Croatia, Italy, South Africa, Sweden suggest rates of 2.3 to 6.3 % among school aged children [21–23]. Other research suggests that higher risk populations including some Aboriginal communities have much higher rates [24, 25]. In a recent systematic literature review and meta-analysis of 33 studies in 9 countries that included Canada, Israel and Sweden, Lange et al., [26] estimated pooled prevalence rates of 16.9 % for FASD and 6 % for ‘full’ FAS. Given this, primary care providers can expect at least 1 %, and likely more (e.g., 2.3–6.3 %), of their patients to have FASD; other professionals (e.g., allied health, education, social services and justice) that serve disadvantaged sub-populations are also likely to work with much higher prevalence rates.

### FASD and the Need for Improved Integration of Care

The 2002 Romanow Commission on the Future of Healthcare in Canada indicated the need for a more comprehensive and collaborative health system [27]. Later in 2013, *the Canadian Institutes of Health Research (CIHR) suggested that the lack of integration of services is a key deficiency in the Canadian health care system* and that examples where improved integration could support the development of a more comprehensive health system included bringing together health and other system stakeholders such as: acute care, first-year care, public health, community care, the educational system, the justice system and parents [28]. There is evidence from primary care settings that quality, health outcomes and provider satisfaction are improved when healthcare systems are more integrated at multiple levels [4, 5].

#### FASD may be one of the best examples of the need for improved integration

It is considered to be underdiagnosed based on both existing prevalence figures (e.g., 2.3–6.3 VS 1 % estimates) as well as existing diagnostic capacity. The physical health and neurodevelopmental disabilities caused by FASD are permanent requiring a lifespan approach to care. In addition, the medical and social complexity that is associated with FASD requires comprehensive assessments, treatments and services from a broad array of medical and non-medical professionals. Examples of medical and non-medical service providers that individuals with FASD may see are indicated in Table 2.

**Table 2:** Selected medical and non-medical professionals providing care to individuals with FASD

Family Physicians	Pediatricians	Developmental Pediatricians
Geneticists	Dysmorphologists	Psychiatrists
Psychologists	Neuropsychologists	Public Health Nurses
Community Nurses	Occupational Therapists	Physical Therapists
Special Education	Family Advocates	Speech Language Pathologists
Social Services	Youth Justice	Probation Officers
Family Therapists	FASD Courts	Surgeons
Employment Counselors	Guidance Counselors	Teachers
Caregivers/Family	Community Leaders/Elders	Non-Governmental Organizations
Nurse Practitioners	Parents	

Recognizing the need for an integrated system to support individuals, families and communities caring for those with FASD, the purpose of this symposium was to bring together key stakeholders to define existing systems, gaps, and research needs. The goal of the symposium was to elicit responses to the research questions and to reach group-specific and multi-stakeholder consensus relevant to the issue of integrated care for individuals with FASD.

## Methods

### Overview

The symposium was held in Winnipeg, Manitoba on October 9, 2014. Participants and presenters were identified in consultation with decision makers and FASD experts with the Healthy Child Manitoba Office (Government of Manitoba), Manitoba FASD Centre, Canada FASD Research Network, Winnipeg School Division, and the Manitoba College of Family Physicians. The main inclusion criteria was that participants were considered to be experts/leaders in their area and had significant experience with FASD. We also wanted representation from specific stakeholder groups that routinely work with individuals and families with FASD. Those attending represented the following stakeholder groups: Allied and Mental Health (*n* = 9); Education (*n* = 6); First Nations & Métis Health (*n* = 2); Parents & Advocates (*n* = 5); Primary Care Physicians/Nurse Practitioners (*n* = 7); Government Health Policy and Canada FASD Research Network (*n* = 12); Regional FASD Coordinators (*n* = 8); Social Services (*n* = 6); and Youth Justice (*n* = 5). Symposium activities were led by a trained facilitator from the Queen’s University Executive Decision Centre who directed sessions by combining the electronic meeting system (EMS) approach with a modified ‘nominal group technique’ (NGT) [29–31]. The University of Manitoba Bannatyne Campus Research Ethics Board decided the project did not require a full review and approval because the project participants were experts in their fields and were responding to policy related questions (UM Ethics Reference Number H214:054).

The symposium had four main sessions: 1) Stakeholder Roles and Introductions; 2) Integration of Care for Individuals with FASD Across Systems; 3) Engaging and Facilitating Partnerships in Delivery of Primary Care: and 4) Information and Research Needs. Each session was designed to build upon the shared information and consensus decisions reached in the previous session. *For all questions except the question on ‘system-level research and information needs’ (e.g., Question 4b), the participants were seated at the stakeholder group specific tables*. In question ‘4b’, participants were randomly assigned to mixed stakeholder group tables. Table sizes ranged from 5 – 11 participants.

### Research questions

- What is our role (our stakeholder group) in caring for individuals with FASD?
- What challenges do you experience in your interaction with other systems in providing care for individuals with FASD across the lifespan?
- What solutions do you recommend for removing or decreasing barriers to integrated care?
- From the perspective of primary care providers/medical organizations, what would help increase integration with other systems?
- From the perspective of non-primary care providers & government organizations, what would help increase integration with primary care providers?
- What are your (stakeholder group’s) most important information and research needs regarding FASD?
- What are our most important system-wide information and research needs?

### Design

#### Pre-workgroup presentations

To provide background information and context, each working session was proceeded by presentations by invited experts who presented on the following: 1) Manitoba’s Provincial FASD Strategy; 2) Integration & Primary Care; 3) Experiences of Families Living with FASD; and 4) Current State of FASD Research in Canada.

#### ‘Modified’ nominal group technique and the electronic meeting system

The process was directed by the facilitator who combined a modified version of the Nominal Group Technique with the Electronic Meeting System (EMS) approach [29–31]. A general overview of the NGT is provided in Table 3. The strength of the NGT as a consensus building technique is the ability to overcome some of the disadvantages normally found with decision making in groups or committees, which are commonly dominated by one individual or by coalitions representing vested interests [29, 30].

**Table 3:** Modified Nominal Group Technique

1. Introduction, presentation to contextualize the issue and the question.
2. The silent phase - Participants seated at tables of 5–8 think and generate individual responses.
3. Item generation phase - Participants at each table present their top five responses.
4. Item clarification - Each table discusses the items on their list and eliminates duplicates.
5. Small group voting and Prioritized List - Each table selects a top 3–10 list which is typed into the Keyboard and displayed on screen to all participants.
6. Large Group Discussion and Consolidated List - All top 3–10 lists generated by the individual tables are displayed onscreen. The facilitator discusses individual responses and works to eliminated duplicate responses and merge similar responses. The result is a non-ranked consolidated consensus list.
7. Large Group Voting and the Multi-Stakeholder Prioritized List – Participants to answer the question: “I*f we could only address five of these in the next year, which ones are most critical*?”

The EMS approach combines expert facilitation with a state-of-the-art group decision support capability and enables groups to generate ideas rapidly and to accelerate the process of consensus building. The system consists of a network of laptops (2–3 per table) accessing software designed to support idea generation, idea consolidation, and idea evaluation and planning. Benefits of the EMS approach include increased: a) structure; b) efficiency (e.g., shorter meeting times); c) participation; and d) generation of ideas/responses (which are all documented automatically) [31]. The EMS approach both complements and augments the NGT [32].

### Data collection and analysis

Collection and analysis of data consisted of four main steps. The first took place prior to the symposium where confirmed participants were asked to forward the research questions to colleagues who were not attending the symposium. Their responses were collated by stakeholder group and were then provided to the appropriate tables to augment the participants’ discussions during the symposium. Step two (e.g., small group responses and consensus) and Step three (e.g., large group consensus and ranking) took place during the symposium. Step four was content analysis. Content analysis is a qualitative data reduction and sense-making approach in which a volume of qualitative material is evaluated to identify core consistencies and meaning [33]. Lists from stakeholder group tables and merged multi-stakeholder group (e.g., large group lists) and top-ranked responses were evaluated to identify emerging themes and to synthesize the main take-home messages. This analysis was conducted in two time periods. First, the facilitator from the Queen’s Executive Decision Centre evaluated the transcripts from the computer generated symposium report. After having conducted hundreds of these events, the facilitator had developed a strong ability to identify themes and issues from the data. Second, and supported by the facilitator’s report, two of the project investigators reviewed all responses generated by participants to confirm or add to the facilitators conclusions.

## Results

### Overview

Participants were seated at stakeholder group-specific tables comprised of 5–11 participants at each table which included: 1)Allied and Mental Health; 2) Criminal Justice; 3) Education; 4) FASD Regional Coordinators: 5) Government & Policy; 6) Parents and Advocates; 7) Primary Care; and 8) Social Services. Their responses to each question were summarized and themes highlighted. Responses to Question #1 are summarized with themes. The top ranked responses to Questions #2- #4 are presented in the following tables and corresponding descriptions of the context and purpose.

### Question 1: What is our role (our stakeholder group) in caring for individuals with FASD?

Stakeholder groups were asked to identify important aspects in their roles as service providers to the FASD population and their families. The purpose of this exercise was to inform the diverse array of professionals in the room and to contextualize the discussions and presentations that followed. Their responses are summarized in Table 4. These results indicate that some stakeholder groups perform a broad range of functions and that across all groups there are similar or shared roles and responsibilities in the following areas: a) education/training; b) advocacy/family support; c) diagnostic, assessments & referrals; d) research or program evaluation; e) coordination/case management; f) program & policy development; and g) direct services provision.

**Table 4:** Stakeholder groups roles

Stakeholder Group	Role in caringfor individuals and families living with FASD
Families & Advocates	1) family advocacy, 2) social services, 3) constant need to educate everyone (e.g., physicians/medical, schools & others).
Primary Care (MDs, NPs)	1) medical (medication/case management), 2) diagnosis, 3) referral, 4) inter-jurisdictional issue resolution (e.g., north vs rural vs urban; provincial vs federal), 5) research, 6) education, and 7) advocacy.
Allied & Mental Health	1) support to pregnant women, 2) parenting/education, 3) prevention, 4) prevention of secondary disabilities, 5) intervention/follow-up, 6) research, 7) comprehensive assessments & diagnostic, 8) mental health diagnosis & confirmation of alcohol exposure, and 9) program planning & evaluation.
Government & Policy	1) providing community funding, 2) policy development/monitoring/updating (e.g. Provincial FASD Strategy), 3) identify priorities & opportunities, 4) training, 5) knowledge sharing (education), 6) creating linkages (e.g., team building), 7) collaboration (multiple levels), and 8) supporting research/evaluation.
Regional Health Authority – FASD Coordinators	1) anchored by the Manitoba FASD Centre, 2) diagnostic (consistency), 3) referrals, 4) assessments, 5) family support, 6) follow-up (services/treatment), 7) program evaluation, and 8) education (multiple groups).
Education	1) educate, 2) develop curricula, 3) meet curricula, 4) provide inclusive & least restrictive environment, 5) life skills, social skills & employment skills, 6) direct services to students, staff & caregivers, 7) advocacy – case management, access funding & services, 8) building teams & sense of community, and 9) training/professional development for multiple groups interacting with FASD.
Social Services	1) eligibility screening (FASD assessment), 2) training, 3) advocacy (families individuals), 4) coordination with other services/providers, 5) case management (link families with resources), 6) provide healthcare services, 7) FASD - program development, and 8) FASD - policy development.
Youth Justice	1) responder to FASD versus a service provider, 2) FASD Youth Justice Program: police, prosecution, defence counsel, probation officer, diagnostic coordinator, 3) focus on purpose in justice system but be aware of issues associated with FASD, 4) Education/Awareness (to multiple groups e.g., police and others), 5) diagnostic services, 6) referrals, 7) coordination with probation services, and 8) follow-up services/resources.

### Question 2a: What challenges do you experience in your interaction with other systems in providing care for individuals with FASD across the lifespan?

The purpose of this question was to identify stakeholder group-specific integration challenges they experienced with coordination and interaction activities across multiple systems. Each of the eight tables were asked to ‘brainstorm’ and submit identified challenges. A total of 130 responses were submitted. The facilitator then directed the tables to discuss and then select their top three most important challenges. The top-three stakeholder group-specific lists are illustrated in Table 5.

**Table 5:** Challenges experienced in interaction with other systems in providing care for individuals with FASD (Top 3 responses)

Primary Care
• No tools for primary care diagnosis and management
• Jurisdictional issues: a) provincial services not available on First Nations Territory; b) multiple health authorities; c) multiple funding sources: d) differing geographical service areas for different services; e) mental health versus medical; & f) lack of trust from communities.
• Mental health systems that work for integration
Parents & Advocates
• Need to have services regardless of diagnosis (behaviours are there and need to be addressed anyway)
• No clear path in accessing services - families are responsible to access and coordinate services
• Constant need to educate everyone we come in contact with (doctors, teachers, etc.) - systems cannot be flexible beyond traditional models
Allied & Mental Health.
• Waiting lists growing with insufficient resources
• Systems saying “I don”t have the expertise to deal with the child with FASD” - using information about FASD as an exclusion criteria; (e.g. daycares, medical/mental health services, programs, schools, etc.)
• Advocacy role for the individual and his or her family in a system that doesn’t share information, in a system where there may be a huge lack of continuity of care
Education
• Keeping kids in school, developing productive contributing citizens…Core curriculum, work experience, life skills, advocacy, core credits for high school
• Privacy/advocacy/gatekeeping/wrap around support/ multiple system contact, little or no communication between systems
• Knowledge base and development of appropriate strategies to support: paradigm shift, reframing, professional development and behavioral strategies
Government & Policy
• Could systems be more adaptive and responsive to people with FASD who don’t fall within usual parameters of programming available?
• System navigation/coordination - hard for families to find what they need, lack of communication between systems
• Needing a diagnosis as a prerequisite to service
FASD Regional Coordinators
• Lack of rural services and services on First Nations Communities
• Length of Waitlist for an assessment
• Program eligibility criteria (e.g., a) mental health ineligible with FASD diagnosis (in some regions); b) Children’s Disability and Community Living IQ 70 or less, school support
Social Services
• Eligibility Criteria/Coordination - Criteria for many services don’t apply to many individuals with FASD (e.g., IQ, etc.) and when individuals are eligible for services the systems are not working together.
• Lack of preventive and supportive services (e.g. respite, in home support, housing, etc.) especially in rural and northern regions. Services that are available are typically set up for short term supports even though families dealing with FASD require services throughout the lifespan.
• Long wait lists for assessments and services
Criminal Justice
• Need for more information on FASD & related disabilities and ability to communicate effectively with individuals with FASD
• Constant need to educate medical and non-medical contacts about FASD
• Limits in Criminal Justice System – require supports from other systems.

In addition to the top-3 challenges submitted by the individual groups, there were common challenges that emerged from the 130 responses submitted by all groups. These included: 1) general lack of awareness of FASD and of services available; 2) waiting lists growing with insufficient resources; 3) culturally appropriate services for First Nations communities; 4) services to individuals over 19 years old; 5) access in northern, rural & remote communities; and 6) inflexibility of some systems and communications across systems.

### Question 2b: What solutions do you recommend for removing or decreasing barriers to integrated care?

Participants at each of the eight stakeholder group tables were asked to ‘brainstorm’ and discuss solutions to the integration barriers and issues identified in the previous question. They were then asked to identify and submit their ‘table-specific’ top-two ideas resulting in 16 responses. The facilitator worked with the whole room to gain consensus and merge similar responses which resulted in a final ‘large group list’ of 11 responses. Then with the objective of obtaining multi-stakeholder (large group) consensus on prioritized responses, the tables were directed to vote on the top 4 ideas from the list of solutions that were the best ideas based upon the decision criteria of having the highest impact and being the most feasible. The resulting multi-stakeholder group ranked list of solutions is illustrated in Table 6.

**Table 6:** Solutions to removing or decreasing barriers to integrated care

Rank	Response/Comment
1	Change eligibility criteria and flexibility to accept individuals with FASD for various programs and service. For example supports in school, community living, mental health and children’s disability.
2	FASD specific system navigator/advocate.
3	Family centred approach to care with case manager support (key workers) - acknowledgement of impact on family - family is the expert - empower families.
4	Create a centralized system for assessment and resources (e.g., housing, support programs, income assistance, health care, child welfare, corrections, and treatment programs). This could be a community based committee.
5	Expanding capacity of regional diagnostic and follow-up processes across the lifespan, including an expanded resource pool for a comprehensive assessment process (e.g. community physicians, school clinicians, etc.), across Manitoba including within Winnipeg (i.e. neighbourhoods). Systems should be interconnected.
6	Create FASD friendly environments in ALL systems - Individuals with FASD accessing services, living in the community.
7	System reform - “my health team” - interdisciplinary primary health care team.
8	Collaborative wrap around approach: more programs and accessibility for after school activities, life coach for the student and family, coordinated system for students as they move through the life span.
9	Some FASD expertise embedded in the criminal justice system to assist with communication, including having specialists in each area, e.g. Probation Services and a resource base for the research associated with FASD and what it really means for the legal assessment of the FASD individual’s status in the system.
10	Information system management -- province wide integrated client record across systems with access by individual/ PC system and linked across systems.
11	Re-evaluating the criteria for an FASD diagnosis (removing the maternal drinking confirmation if other indicators strong), including programs and resources for those who meet the criteria but don‘t’ have the actual diagnosis.

### Question 3: From the perspective of primary care providers/medical organizations, what would help increase integration with other systems?

This question and the following question were intended to address statements from the Romanow Commission on the Future of Healthcare in Canada and the Canadian Institutes of Health Research related to the need for a more comprehensive and collaborative health system. Primary care is often the place of first contact and is well positioned to support integrated care. The purpose of this session was to identify ways to increase collaboration/integration in this context. *This question was answered only by the Primary Care table.* The most important approaches to helping increase integration identified by the Primary Care table are illustrated in Table 7.

**Table 7:** From the Perspective of Primary Care Providers, what would help to increase integration with other systems?

**Develop a FASD Tool Kit for Primary Care**
Disseminate the Tool Kit using Knowledge-to-Action principals to ensure uptake. (Tool Kit for: diagnosis, referrals, treatment and behavioural management). Also, develop a primary care FASD APP based on the FASD Tool Kit.
**Develop/Implement an Expert hotline**
(e.g., analogous to UPCON - uniting primary care and oncology)
**Patient virtual passport - Integrated Electronic Record System**
(Include client’s resources for education support, mental health support, medical experts. It should be accessible to the client and family centred.)
**Clear role definition for the members of a person’s care team**
(Could we have a template for the key components of a care team - FASD care plan?)
**Clients need to be able to identify their primary care home**
(E.g.,. people often answer: “I don”t know” when asked who is their primary care provider. Could the system link people to the most appropriate primary care home?
**Reports and Assessments need to go to primary care provider**
**Develop/Add FAQ - pages for parents and primary care providers on the FASD website**
**Tool kit for parents**

### Question 3b: From the perspective of non-primary care providers & government organizations, what would help increase integration with primary care providers?

This question was answered by the non-primary care stakeholder tables. These seven tables were asked to ‘brainstorm’ to develop table-specific suggestions to increasing integration with primary care. This activity generated 40 responses. Tables were then instructed to reach consensus on their Top-2 ideas and to submit them to the room. The facilitator then worked with the room to merge similar responses from the table-specific Top-2 lists. This reduced the total number of responses and resulted in a prioritized list of responses from the ‘non-primary provider’ tables. This list is illustrated in Table 8.

**Table 8:** From the Perspective of ‘Non-Primary Care Providers’, what would help to increase integration with other systems?

**Information Integration/Sharing: there is a need for coordinated and open communication between all stakeholders **(e.g., case worker, family, doctors & mental health workers etc..) Privacy is a huge barrier. Need to re-evaluate privacy policies and eliminate barriers for optimal care while maintaining human rights. Is there a better way to facilitate access, sharing of information? Sharing information needs to have an umbrella release, (e.g., automatic sharing of information between all parties and to make the process faster and efficient).
**Implement a Specialist “medical home” **- where primary care providers are integrated with the medical home team. Could use nurse practitioners as a form of liaising with primary care.
**Introduce a follow-up/outreach responsibility to primary care **system serving vulnerable populations (FASD).
**Provide training and consultation about FASD to physicians** (E.g., similar to the FASD specialist roles within CFS and/or utilize existing coordinators in the five regions to facilitate trainings.
**Increase primary care provider’s knowledge **of possible impairment associated with the specific disability and/or which resources to contact to request information.
**Shared care model**: consultation models/systems for primary care providers to access expertise of their colleagues at a specialist clinic/service. It would be part of the role of the specialist to have dedicated time in their job description to offer consultation.
**Obtaining qualitative information regarding what prevents primary health care providers from making a referral for an FASD assessment**.
**Language** - All talking the same language that’s understandable, communication, medical jargon is too much, shared language, very specific to the needs of the individual.
**Increase diagnostic capacity**. Do this by recruiting primary physicians who are interested in becoming diagnosticians in FASD.
**FASD Medical Access Centre **- This would be a physical space/clinic) that is flexible in approach to services and would be adapted to FASD behaviours/characteristics and other health needs.

Notable responses that did not make the Top Ranked list in Table 8 include: 1) case managers embedded into the primary care system; 2) have physicians attend meetings (or attend by phone) whenever possible; 3) allow a FASD support person to access medical appointments/procedures and meetings; 4) specialized workers who are system navigators; 5) provide screening and brief intervention tools to primary care; and 6) primary care should use culturally appropriate practice when working with diverse populations.

### Question 4a: What are your (stakeholder group’s) most important information and research needs regarding FASD?

The purpose of this question was to provide background information and context for the next question on ‘system-wide’ research priorities. The objective was to identify the most important information and research needs that the different stakeholder groups identified were critical in order for them to be more effective in their jobs. The ‘Top 3’ most important stakeholder-specific information and research needs were presented to the whole room to inform the different groups about the multi-stakeholder research/information needs. These are illustrated in Table 9.

**Table 9:** Most important information and research needs

Primary Care
• Is there an association between cultural continuity (Chandler and Lalonde) and rates of FASD?
• If communities re-establish cultural practices/knowledge, do the rates of FASD change?
• Effective Therapies: a) What non-medical therapies are effective for FASD? (e.g., neurorehab, exercise, meditation). b) How to take advantage of neuroplasticity? c) RCTs to evaluate medical treatment for behaviour.
Parents & Advocates
• Knowledge Translation: Best practices to bring research knowledge to service provision and families.
• Root Causes, Impacts and Prevention: Poverty, racism, colonialism, marginalization, demoralization, stigmatization.
• Why Manitoba has so many children in foster care?
Allied & Mental Health
• Identification of early factors that are indicative of later functioning.
• What is the knowledge base of various community professionals that we interact with and what are the gaps in knowledge?
• What are the outcomes of a diagnostic assessment? What are the impacts over time?
Education
• Low enrollment versus integration/streaming and the impact of secondary disabilities.
• Best Practices: In early years, middle years and high school
• Best Practices: For keeping students in school, graduating, and leading a productive life.
Government & Policy
• What are the protective factors for individuals with FASD that influence stability? (e.g., environmental, lack of trauma, degree of brain injury, resiliency,..)
• Would integrated care for women with FASD assist with FASD prevention?
• What are the most effective intervention strategies for youths and adults?
FASD Regional Coordinators (Diagnostic Network)
• Longitudinal Study: What is the quality of life of adults who received or had a DX of FASD made while they were in the care of child welfare?
• How many children diagnosed with FASD have a diagnosis of attachment disorder? (Or have risk factors for attachment disorder?)
• Best Practices: For providing optimal care/treatment for adolescents with mental health needs/services involvement.
Social Services
• Research on the impact of a diagnosis on families and communities (e.g., readiness, challenges & benefits).
• Functional Evaluation Research: E.g., compare & contrast the functioning of individuals with IQs under 70 with individuals diagnoses with FASD.
• Are there alternative tools to measure adaptive/functional skills for individual with FASD
Criminal Justice
• Greater specificity about the impact of FASD on a particular behaviour of individuals.
• Who are the ‘experts’ for potential Court testimony?
• Statistics relating to the prevalence of FASD in the criminal justice system.

Notable responses that did not make the Stakeholder Group’s Top-3 list in Table 9 include: 1) who does and does not access diagnostic services and what is the difference between groups; 2) best practices/prevention – to keep individuals off/away from the ‘streets’; 3) the need for better prevalence data in different populations (e.g., foster care, incarcerated/justice system, socioeconomic & ethnic/culture; 4) need for a biological test/marker; 5) best practices (e.g., occupational therapy, speech language pathology) and medical treatment for pre-school individuals with FASD; 6) develop or identify what psychological assessment tool is most effective at informing treatment/intervention planning; 7) risk assessment tool (e.g., to identify those at higher risk of criminal activity/offending); and 8) are there cost savings that result from integrated care models?

### Question 4b: What are our most important system-wide information and research needs?

The purpose of this question was to obtain multi-stakeholder consensus on ‘system-wide’ research and information needs. The symposium participants were asked to think of the ‘system as a whole’ versus from the perspective of their stakeholder group.

The Canadian Institutes of Health Research (CIHR) – Institute of Human Development, Child and Youth Health provided funding for the symposium. The institute indicated that: a) forward-thinking, creative and innovative solutions are absolutely essential for the future of our health care system; and b) the lack of integration of services has been identified as a key deficiency in the Canadian health care system. CIHR also recognized that one step supporting the move towards better integration was to identify and reach consensus on research needs, gaps and opportunities [28].

To facilitate this activity, participants were randomly re-assigned to new mixed stakeholder tables. Then the facilitator directed all the new groups to brainstorm and discuss system-level information/research needs. Each group shared its top two ideas (onscreen). All responses were then clarified and merged (e.g., similar responses) by the facilitator resulting in the final ‘large group’ system-level list which was then voted on to generate the ranked list of system-level research and information needs illustrated in Table 10.

**Table 10:** Top 10 System-Level Information and Research Needs

Rank	Response/Comment
1	**New Models of Child & Family Support **- E.g., “healthy village” around the child. How can we support mothers/families at risk? Can we minimize putting children in care by providing alternate supports?
2	**More Longitudinal Outcome Data **- Post diagnosis: how are children/adults doing? How did a diagnosis impact their lives? What are the factors that affect positive and negative outcomes? What is the effect of aging (e.g. adolescence, older adults) on the support needs of individuals with FASD? Do we need an influx of services at certain times vs. a consistent level of support throughout the lifespan?
3	**Quality of Life & Supportive Services for Parents/Caregivers **- What is the impact on the parent’s/caregiver quality of life across the life span? What are the best practices for supporting parents of children with FASD? (e.g., supports for parent well-being and supports in their parenting role.)
4	**Cost-Benefit or Minimization Analysis **- What would be the cost savings to the system if an individual with FASD had full access to required supports (e.g. filling the gap…adequate housing, mentoring, physician, mental health, etc.)?
5	**Community-Based Participatory Research Methods **- How do we engage in community directed research with the First Nations communities?
6	**Family-based Needs Analysis **- Qualitative study of family needs. What have they found useful; suggestions for what their needs are and what would help them navigate the system better/what have they found successful.
7	**Resiliency/Protective Factors**: What are the protective factors that result in improved quality of life for a person with FASD?
8	**Intervention Research **- Effective intervention strategies - what works, what are the best options? What are solid life skills that a child needs in order to be successful (i.e. cooking, money management)? What are the pragmatic skills that everyone needs?
9	**Reducing/Minimizing Effects of Alcohol in Pregnancy **- What works to minimize effects of alcohol exposure in pregnancy -- harm reduction e.g. nutrition, prenatal care, other factors; what positive supports make a difference
10	**Integration & Best Practices **- What model works best for integration of services?

## Discussion and conclusion

FASD is a complex neurodevelopmental disorder that results in permanent disabilities. It is described as a ‘hidden disability’ because there are no overt physical signs to identify an affected child. Various and challenging behaviours are often the only symptoms in this ‘brain based disability’ which recent evidence suggests may affect 2–6 % of the population [20]. The increased rate of cognitive disability along with academic disruption, as well as neurobehavioural and mental health disability, and often layered social complexity results in a significant need for health, social and community services.

Research also suggests much higher rates in at-risk sub-populations. For example, children with FASD are much more likely to ‘drop out’ of school or be suspended, both of which have been shown in Canadian and American studies to be risk factors for involvement in crime and subsequently the criminal justice system [34]. Research has demonstrated that individuals with FASD form the largest group of young people entering the criminal justice system [3]. For example, in their systematic review of Canadian data, Popova et al., reported that in a given year youths with FASD were 19 times more likely to be incarcerated than youths without FASD [35]. The United States Department of Health and Human Services reported a Washington study which estimated that 35 % of individuals with FASD had been in jail or prison and that more than 50 % had been in trouble with the law [36]. These individuals are impacted by ‘secondary disabilities’ associated with FASD that include mental health issues, school withdrawal, legal problems and substance abuse all of which are compounded by a lack of community and societal understanding and accommodation [11, 37]. These individuals are often identified later in life or prior to an optimal ‘best time’ for interventions that could effectively address the effects and secondary disabilities of FASD. Better integration, including better communication and collaboration across systems, would serve to decrease the impact of FASD on both affected individuals and the general population.

These arguments are supported by Bredberg, an education consultant who visited 460 schools in British Columbia with children diagnosed with FASD. Bredberg indicated that professionals involved in the care of children with FASD were not getting the message across adequately to education professionals that there are neuro-behavioural differences in FASD compared to other learning disabilities. She suggested that it is imperative to better integrate findings from multidisciplinary diagnoses into educational programs. In a process described as bi-directional capacity building she said:

*“Not only can education practice be informed by multidisciplinary diagnoses, but it was our observation that diagnostic practice can be informed and enlightened by hearing from education.”* (Bredberg cited in Jonsson et al., [38], p. 123)

The issue of stigma or negative stereotyping, and resultant discrimination, can also be reduced by comprehensive efforts at system integration. The perspectives of a broad array of system stakeholders (e.g., health and non-health service providers and policy makers) that are centred on affected individuals and families, are necessary to designing an ethically grounded, responsive, and well-integrated system. In our symposium, both new models of child and family support as well as community-based participatory research methods were identified among the top-10 system level information and research needs (see Table 10). It has been further suggested that the broad array of medical and non-medical providers across systems who work with FASD (see Table 1) are to various degrees interdependent. Thus, improving integration through engaged and thoughtful community based partnerships in prevention, diagnosis, treatment, support, intervention, and research at individual, community, and policy levels will increase both provider-specific and overall system capacity. This increased capacity will translate into: 1) a decreased impact of FASD (e.g., human health, economic and secondary disabilities); 2) an increased quality of life for individuals and families living with FASD, and 3) increased provider satisfaction.

### Implications

The findings of this symposium represent consensus decisions reached by a diverse multi-stakeholder group of medical and non-medical service providers with years of expertise in FASD including leaders with FASD expertise from the following groups: i) Allied and Mental Health, ii) Education, iii) First Nations and Métis Health, iv) FASD Regional Coordinators, v) Government and Policy, vi) Primary Care (MDs and NPs), vii) Social Services, and viii) Youth Justice.

The main implications of our findings include:

- There is a clear need to better integrate care for individuals and families living with FASD. This is related to the complex nature of FASD and the interdependency of the broad array and number of medical and non-medical providers who work with FASD across the lifespan.
- Primary Care is well positioned to play a central and important role in facilitating and supporting increased integration. This is not a primary care only responsibility. However, primary care is best positioned to facilitate the integrated care (e.g., assessment, referrals, treatment, coordination and communication) across systems.
- Approaches that would support increased integration of care include: a) electronic medical records; b) a client centered medical home; and c) multidisciplinary primary care group practice models. Increased information sharing among both medical and non-medical service providers through eHealth will increase efficiency and system-level capacity. A client centered medical home that includes client/family advocates to help navigate the system will increase satisfaction and outcomes. Group practices will increase provider capacity within group and both efficiency and communications.
- Main barriers to integration include: a) eligibility criteria for services; b) inadequate access to services in rural and remote areas; c) lack of system-level awareness, knowledge and capacity; d) lack of culturally appropriate approaches in First Nations communities. Eligibility criteria do not equitably address the disabilities and service needs of FASD compared to other disorders.
- Research is needed to better address best practices (e.g., interventions, supports and programs) and long-term individual and family outcomes following a diagnosis of FASD.

### Limitations

All participants at the symposium were from Canada with the majority coming from the province of Manitoba. We recognize that health systems and the degree of collaboration between multiple systems (e.g., health and non-health) will differ between provinces and countries. Consequently, all of the findings and policy suggestions we report cannot be generalized to other locations. However, in all locations where alcohol is consumed there very likely will be FASD which is a permanent and complex disability that will be better addressed with increased awareness and integration. Given this, we believe many of the findings will be relevant to readers outside of the province of Manitoba.
